# Serrated Colonic Polyps in a Teaching Hospital in Saudi Arabia: Prevalence and Review of Classification

**DOI:** 10.4103/1319-3767.56097

**Published:** 2009-10

**Authors:** Rana Bokhary

**Affiliations:** Department of Laboratory Medicine, Anatomical Pathology, King Abdul Aziz University Hospital, P.O. Box 80215, Jeddah 21589, Saudi Arabia

**Keywords:** Dysplastic serrated polyps, non-dysplastic serrated polyps, serrated colorectal polyps

## Abstract

**Background/Aim:**

To determine the prevalence of serrated colorectal polyps in the King AbdulAziz University Hospital population and to review the current classification of colorectal serrated polyps with emphasis on morphological features.

**Materials and Methods:**

This retrospective study used cases diagnosed with serrated colorectal polyps at the histopathology laboratory of King AbdulAziz University Hospital during last five years (2004-2008). The slides were reexamined microscopically and the lesions were renamed according to the terminology discussed in this article.

**Results:**

Diagnosed hyperplastic polyps represented 12.3% of all colorectal polyps submitted to our laboratory during the study period. However, the false positive rate was found to be 42.5%. Of the truly diagnosed serrated polyps, the most common subtype is the microvesicular serrated polyps. The majority of the serrated colorectal polyps was found in males, with a wide age range.

**Conclusion:**

The prevalence of serrated colorectal polyps in our geographic area seems to be similar to that in western populations.

Serrated colorectal polyps, once considered a homogenous group, are a heterogeneous group of mucosal gastrointestinal lesions that include the commonly diagnosed “hyperplastic” polyps. It is important for both pathologists and clinicians to recognize the relatively newly described different subtypes of serrated colorectal polyps as some of them are considered premalignant. These need to be completely resected and the patient should be followed-up more closely.[1,2] The prevalence of serrated colorectal polyps was studied previously in different western populations, but its prevalence in Arab world is not clear although they are seen frequently in our histopathology laboratory.[3–7]

## MATERIALS AND METHODS

The study cases were drawn from the King AbdulAziz University Hospital Pathology Laboratory archives, which is one of the largest hospitals in the western province of the country. These comprise of cases that were diagnosed as “hyperplastic” polyps in the last five years (January 2004 - December 2008). The slides of these cases were re-examined microscopically and the cases subclassified according to the latest pathological nomenclature for the serrated colorectal polyps discussed below to evaluate their prevalence relative to other types of biopsied or resected gastrointestinal polyps and to evaluate the prevalence of the different subtypes. Simple descriptive statistics were used to analyze the data.

## RESULTS

Over the studied five years period (2004-2008), a total of 325 colorectal polyps were resected and sent to our laboratory. Only 40 of these polyps were diagnosed as serrated polyps (12.3%). Of these, 29 were diagnosed as hyperplastic polyps, 6 as mixed adenomatous and hyperplastic polyps, 2 as serrated adenomas, 1 as sessile serrated polyp, 1 as hyperplastic pseudoinflammatory polyp and 1 as hyperplastic polyp with features of mucosal prolapse. Reexamination of the microscopic slides showed only 23 (57.5%) of the originally diagnosed serrated polyps to have a true serrated morphology, while 17 (42.5%) of these cases were mislabeled as serrated polyps. These falsely mislabeled polyps were only found in the cases originally diagnosed as hyperplastic polyps. These were found to be either tubular adenomas (seven cases), mucous retention “juvenile” polyp, mucosal prolapse-type polyp, colonic xanthoma, reactive lymphoid hyperplasia, chronic quiescent colitis in a known case of inflammatory bowel disease, active colitis (two cases) or normal bowel mucosa (three cases).

Of the truly diagnosed serrated polyps, the majority was found to be of the microvesicular subtype (12 cases = 52.2%). The next most common subtype is the conventional adenoma with serrated architecture (6 cases = 26.1%). There were three goblet cell serrated polyps (13%) and two unclassifiable cases. One of the latter two cases was without dysplasia that could not be classified because of extensive surface ulceration. The other was with dysplasia but its features did not fit with the described features of any of the dysplastic serrated polyps. There were no cases of sessile serrated polyps, mucin-poor serrated polyps or serrated adenomas.

The age of the patients re-classified with true serrated polyps ranged from 29 to 75 years. A majority of the patients were males (M:F = 4.75:1).

## DISCUSSION

Serrated colorectal polyps are a heterogeneous group of mucosal gastrointestinal lesions. Although they are highly prevalent in western populations, their prevalence is not clear in Arab region.[3–7] Specific lifestyle and dietary factors appear to be associated with increased prevalence of hyperplastic polyps, such as cigarette smoking, alcohol consumption, obesity and low folate intake.[8–10] Other factors such as non- steroidal anti-inflammatory drugs (NSAIDs) intake, high calcium intake and hormone replacement therapy were found to reduce the risk of their occurrence.[9] Until recently, they were considered entirely harmless and unlikely to progress to carcinoma. The study of hyperplastic polyposis showed that some variants of hyperplastic polyps can progress to colorectal carcinoma and unveiled the third pathway of colorectal carcinogenesis, which was called serrated neoplasia pathway.[11–16] These studies led Torlakovic and coworkers to study the morphological growth pattern of sporadic hyperplastic polyps, leading to the identification and description of the different histological subtypes of serrated colorectal polyps.[17]

### Morphological futures of serrated colorectal polyps

**Figure 1:**
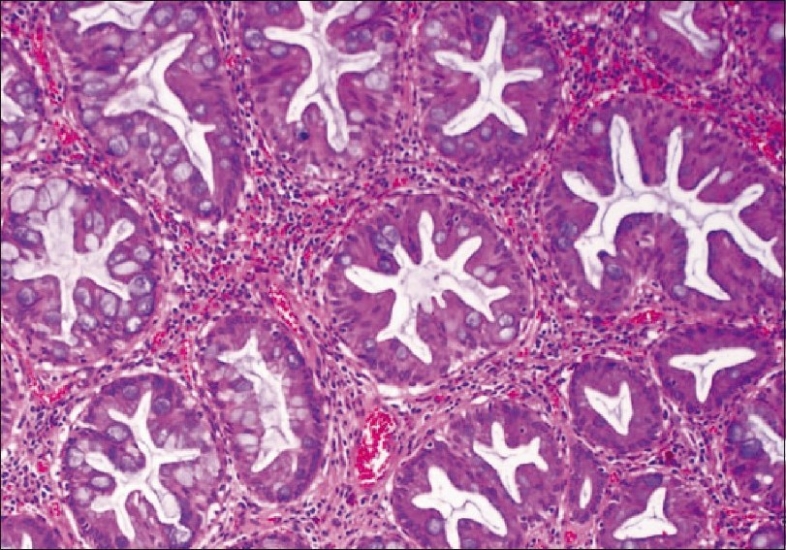
Cross section of crypts revealing stellate appearance resulting from epithelial infolding (H and E stain)

Before describing the morphology of the different subtypes of serrated polyps, the meaning of serration should be explained. All serrated polyps should show some degree of infolding of the crypt epithelium, which leads to the characteristic saw-toothed appearance in longitudinal sections and the stellate appearance on cross-sections of the crypts [Figure 1].

### Nondysplastic serrated polyps

#### Polyps with normal architecture and proliferation

These polyps are usually of small size (<5 mm) and approximately 90% of them are found incidentally in the rectosigmoid colon.[18] The hallmark of these polyps is that the lower third of crypts remain narrow. The crypts gradually become dilated toward the upper half or third of the mucosa and serration is most pronounced in this dilated part. Recently, some authors have proposed three additional subtypes of nondysplastic hyperplastic polyps with normal architecture and proliferation based on degree of serration, content of cytoplasmic mucin, and the number of goblet cells: Microvesicular type, goblet cell–rich type and mucin-poor type.[17] The microvesicular type is the most common type and there is some evidence that this type may rarely progress to carcinoma.[12,18,19] Therefore, general surgical pathologists may need to be aware of this subclassification as it might be clinically significant.

**Figure 2:**
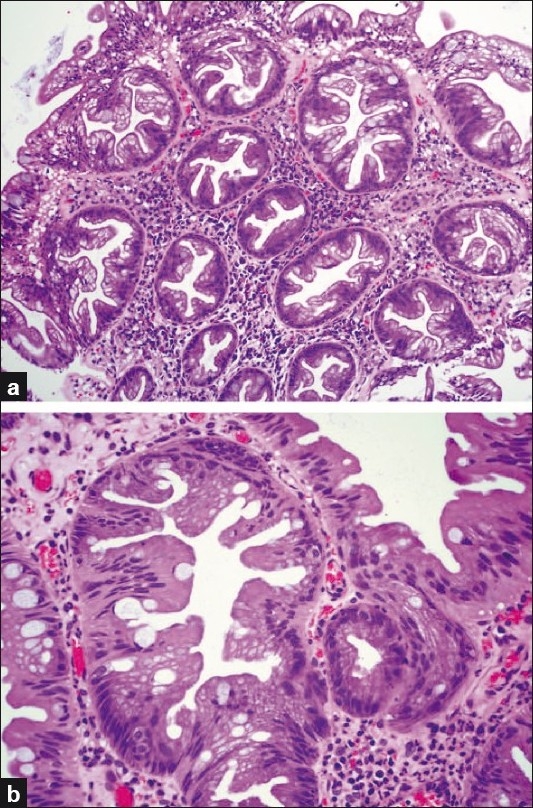
Microvesicular serrated polyp; (a) Low power view showing prominent crypt serration; (b) High power view of a crypt which is typically lined by enlarged microvacuolated cells and few goblet cells. Some degree of nuclear stratification can be seen in these polyps (H and E stain)

The microvesicular serrated polyps are characterized by the presence of abundant enlarged microvacuolated columnar cells admixed with small number of goblet cells in comparison with the normal mucosa [Figure 2]. The serration is prominent and confined to the upper portion of crypts. Although some of them might show mild degree of nuclear stratification and atypia, these features are limited to the lower part of the crypts, and there is a clear cut evidence of surface maturation. Mitoses are rare and seen only in the base.

**Figure 3:**
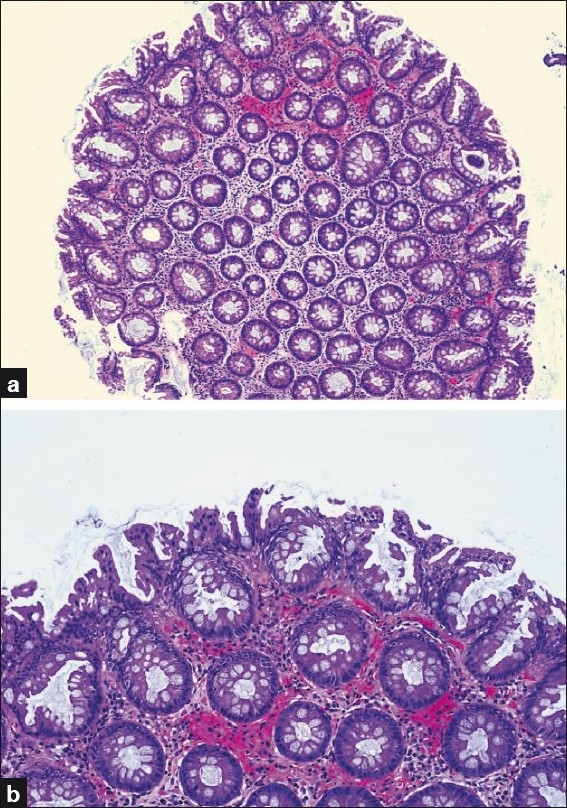
Goblet cell-rich serrated polyp; (a) Serration in this subtype is not prominent and is limited to the upper parts of crypts as demonstrated in this low power view; (b) High power view showing prominent goblet cells and characteristic surface serration and tufting (H and E stain)

Goblet cell-rich serrated polyps are the second most common subtype. They show elongated crypts with abundant goblet cells but no microvacuolated cells [Figure 3]. Serration is far less prominent than microvesicular subtype or even absent, but tufting of surface epithelium, which is also rich in goblet cells, is characteristic.[17] Nuclear atypia, stratification and mitoses are not seen in this subtype.

Mucin-poor serrated polyps are the least common subtype. The cells lining the crypts are small, with scanty cytoplasm and almost no mucin imparting a regenerative look. They do not have goblet cells and have a micropapillary architecture. Some investigators believe that they are microvesicular polyps that have undergone irritation and inflammation as a result of injury.

#### Polyps with abnormal architecture and proliferation

Although sessile serrated polyp crypt epithelium shows no overt conventional cytologic dysplasia, it was called in the past a sessile serrated adenoma. The “dysplasia” in this type of polyps resides in its abnormal unique architectural pattern and proliferation. They are right-sided lesions that are usually large (>5 mm) and sessile. If resected completely with the adjacent mucosa, they are seen as slightly elevated lesions. However, this feature may not be obvious on biopsy samples due to contraction artifact and/or lack of normal adjacent mucosa.[20] On low power magnification, they show crypt architectural distortion and asymmetry. Crypts often appear flask-shaped, with characteristic dilatation and branching of their bases parallel to muscularis mucosa, creating inverted T- or L-shaped crypts. Serration extends from the surface to base of the crypts. The crypts both at the base and on surface contain mature columnar cells, goblet cells and cells with gastric foveolar cell phenotype. Focal nuclear stratification may be seen either at the base or on the surface. Nuclei generally are small and hyperchromatic, without open chromatin or nucleoli. The nuclear-cytoplasmic ratio is lower than that of conventional adenomas. Mitoses are not as numerous as in conventional adenomas, although mitoses usually are present in both throughout crypts.

### Dysplastic serrated polyps

Similar to conventional adenomas, *conventional serrated adenomas* should have uniform, overt cytological dysplasia; however, they are characterized by confluent, eosinophilic cytoplasm and papillary or villiform growth pattern.[20] There is much confusion in the literature in regard to the dysplastic serrated polyps in general and the serrated adenomas in particular, as many serrated dysplastic and nondysplastic polyps have been named as serrated adenomas; therefore, the clinical picture, epidemiology and natural history of the conventional serrated adenomas are poorly understood and need to be studied further. However, there are some studies indicating that this uncommon type of polyps occur in older individuals than population with nondysplastic serrated polyps and that they occur more commonly in females and exclusively in the left colon.[18,21,22]

**Figure 4:**
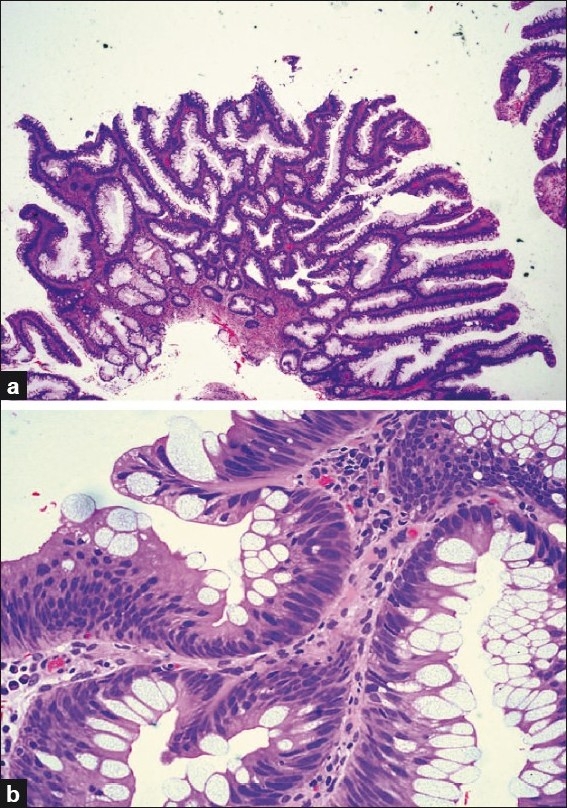
Conventional adenoma with serrated architecture; (a) Low power view revealing abnormal crypt architecture; (b) Elongated, cigar-shaped nuclei which are stratified and hyperchromatic (high power view) (H and E stain)

Some *conventional adenomas* acquire *serrated architecture* and need to be distinguished from the conventional serrated adenoma by their nuclear features that appear exactly like the nuclei of conventional adenomas. The nuclei are cigar-shaped, have clumped chromatin and exhibit stratification [Figure 4].

Over time, some sessile serrated polyps progress and develop dysplastic foci. These polyps were called mixed hyperplastic/adenomatous polyps in the past. But as we know now that these polyps do not represent a coincidental occurrence of hyperplastic and adenomatous changes in the mucosa but result from molecular changes and progression in sessile serrated polyps, leading to morphologic change of dysplasia, they should be named *s*essile serrated polyps with dysplasia.[12,19] The dysplastic foci resemble either conventional adenomas or conventional serrated adenoma, or they may show combined features of both subtypes.

If it is not possible to fit the serrated polyp into one of the above described subtypes, these polyps can be called unclassifiable serrated polyp with/without dysplasia. If unable to fit the serrated polyp into one of the above described subtypes, then they can be called unclassifiable serrated polyp with/without dysplasia.[23]

## CONCLUSION

Serrated polyps are the second most common type of polyps arising within the large intestinal mucosa and their prevalence in Saudi population appears to be similar to western population. As some of their subtypes may progress to invasive carcinoma, pathologists should be aware of their current classification and nomenclature. They also should be able to differentiate them from their mimickers, especially mucosal prolapse-type polyps. As their classification is becoming clearer, studying the natural history of each subtype is required to establish more guidelines for treatment and surveillance of patients.
